# Multieffect Coupled Nanogenerators

**DOI:** 10.34133/2020/6503157

**Published:** 2020-12-16

**Authors:** Yun Ji, Yuan Liu, Ya Yang

**Affiliations:** ^1^CAS Center for Excellence in Nanoscience, Beijing Key Laboratory of Micro-Nano Energy and Sensor, Beijing Institute of Nanoenergy and Nanosystems, Chinese Academy of Sciences, Beijing 100083, China; ^2^School of Nanoscience and Technology, University of Chinese Academy of Sciences, Beijing 100049, China; ^3^Center on Nanoenergy Research, School of Physical Science and Technology, Guangxi University, Nanning 530004, China

## Abstract

With the advent of diverse electronics, the available energy may be light, thermal, and mechanical energies. Multieffect coupled nanogenerators (NGs) exhibit strong ability to harvest ambient energy by integrating various effects comprising piezoelectricity, pyroelectricity, thermoelectricity, optoelectricity, and triboelectricity into a standalone device. Interaction of multitype effects can promote energy harvesting and conversion by modulating charge carriers' behaviour. Multieffect coupled NGs stand for a vital group of energy harvesters, supporting the advances of an electronic device and promoting the resolution of energy crisis. The matchless versatility and high reliability of multieffect coupled NGs make them main candidates for integration in complicated arrays of the electronic device. Multieffect coupled NGs can also be employed as a variety of self-powered sensors due to their rapid response, high accuracy, and high responsivity. This article reviews the latest achievements of multieffect coupled NGs. Fundamentals mainly including basic theory and materials of interest are covered. Advanced device design and output characteristics are introduced. Potential applications are described, and future development is discussed.

## 1. Introduction

Scavenging of chemical, light, wind, and thermal energies is a long-term and vibrant topic with the fast growth of electronics as well as the rising requirements for green energies [1–4]. Energy harvesters ushered in their new era since Wang and Song developed a nanogenerator (NG) on the basis of a single ZnO nanowire (NW) in 2006 [5]. Subsequently, a variety of NGs have been developed, including thermoelectric NG (ThENG), pyroelectric NG (PyENG), piezoelectric NG (PNG), and triboelectric NG (TENG) [6–10]. Although showing remarkable performance, these NGs and conventional photovoltaic devices (PVCs) are only available in a specific ambient circumstance relying on variable factors comprising weather and location. Multieffect coupled NGs integrate various mechanisms of electricity generation, including pyroelectric, piezoelectric, triboelectric, photovoltaic, and thermoelectric effects into a single unit, making them possible to harvest whatever energies are available anytime [11–14]. Multieffect coupled NGs can not only offer a sustainable electricity supply by scavenging clean and renewable energies but also contribute to maximizing energy collection to obtain steady and high electric output [15]. Interaction between various effects of electricity generation can modulate the production, separation, transport, and recombination of carriers and finally influence the multieffect coupled NGs' energy conversion [16, 17]. As important members of energy harvesters, multieffect coupled NGs support the rapid growth of electronics and help address energy crisis issues. With the development of intelligent life, a variety of complicated self-powered electronic device arrays will be utilized everywhere in the future. The matchless versatility, high reliability, and miniaturization of multieffect coupled NGs make them main candidates for integration in the complicated electronic systems [18]. Additionally, for multieffect coupled NGs, their performance strongly depends on the type and strength of external stimuli, and their rapid response, high accuracy, high selectivity, and high responsivity to various stimuli make them promising candidates for a variety of self-powered sensors [19, 20].

This paper reviews multieffect coupled NGs with a detailed look at their fundamentals and most recent advancements. The review starts with a brief introduction of fundamentals for multieffect coupled NGs, including basic theory of thermoelectricity, pyroelectricity, triboelectricity, optoelectricity, and piezoelectricity, development process, and materials of interest. And then, an overview of the state-of-the-art device configuration is presented. Subsequently, output characteristics, with special emphasis on output current and output voltage, are described. And then, some of the recent applications mainly focusing on enhanced light harvesters, multienergy harvesters, self-powered photodetectors, self-powered multifunctional sensors, and self-powered image sensors are introduced. Finally, future development is discussed.

## 2. Fundamentals

NG is a nascent technology which generally utilizes nanomaterials to scavenge thermal, mechanical, and light energies in the ambient circumstance to produce electricity. NG was first presented in 2006 [5]. The working principles of NGs are on the basis of pyroelectric, piezoelectric, photovoltaic, thermoelectric, and triboelectric effects of semiconductors and ferroelectrics.

### 2.1. Basic Theory

#### 2.1.1. Pyroelectric Effect and PyENG

**Figure 1:**
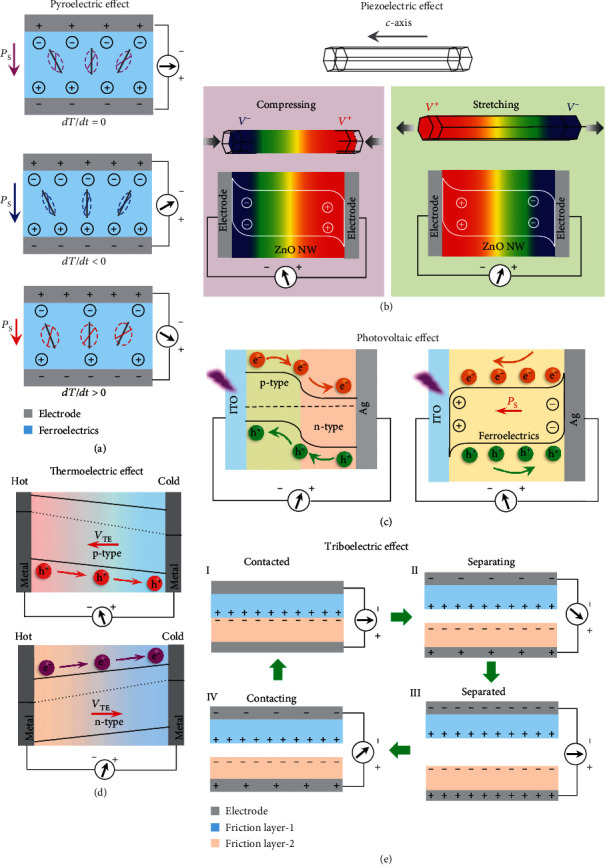
Basic theory for multieffect coupled NGs. (a) Pyroelectric effect in ferroelectrics. (b) Piezoelectric effect in a single ZnO NW. (c) Photovoltaic effect in p-n junction and ferroelectrics. (d) Thermoelectric effect in p-semiconductor and n-semiconductor. (e) Triboelectric effect in a contact-separation type TENG.

Materials can be divided into polar and nonpolar materials according to their average dipole moment. The dipoles offset each other owing to symmetrical structures of the nonpolar materials. However, in polar materials, the dipole moments cannot offset each other due to the noncentrosymmetric structure and show a spontaneous polarization, resulting in remarkable physical properties, such as pyroelectricity. Pyroelectric effect results from a variation of polarization strength with changes in temperature. PyENG which can convert thermal fluctuation into electricity is developed based on the pyroelectric effect of polar materials. The first PyENG was constructed on the basis of ZnO NW semiconductors in 2012 [21]. Subsequently, PyENG based on ferroelectric KNbO_3_ materials was realized [22].  Figure 1(a) demonstrates the working principle of a ferroelectric-based PyENG, where a ferroelectric material is sandwiched between electrodes. Positive and negative charges are attracted to the top and bottom electrodes for screening polarization, respectively. Cooling (*dT*/*dt* < 0) the device will reduce the oscillation of the dipoles and thus promote the polarization strength. To screen the enhanced polarization, the compensation charges increase, resulting in pyroelectric current signals moving towards the top electrode. On the contrary, applying a heating process (*dT*/*dt* > 0) on the device can increase the electric dipoles' oscillation, leading to a lower polarization strength. As a result, reverse pyroelectric current is created.

#### 2.1.2. Piezoelectric Effect and PNG

Piezoelectric effect is a synergy of mechanics and dipole moments and observed in materials with noncentrosymmetric architecture, including some wurtzite single crystals and ferroelectrics [23, 24]. With strains acting on piezoelectric materials, relative displacement between the centers of cations and anions of the piezoelectric materials is created. Consequently, the piezoelectric polarization in piezoelectric materials is changed, leading to piezopotential. A PNG is realized based on the piezopotential and can be employed to harvest mechanical energy.  Figure 1(b) demonstrates the generation process of piezoelectric signals in a PNG based on single ZnO NW. When the ZnO NW is compressed, piezoelectric positive potential and negative potential are created at the -*c*-axis extremity and +*c*-axis extremity, respectively. Under short-circuit condition, negative and positive charges can be attracted to the -*c*-axis extremity and +*c*-axis extremity, respectively. Consequently, piezoelectric current is generated and flows from the -*c*-axis extremity towards the +*c*-axis extremity. When the ZnO NW is released, the piezopotential gradually vanishes and the attracted charges return back, resulting in reverse piezoelectric current. On the contrary, when the ZnO NW is stretched, piezopotential which points from the +*c*-axis end to the -*c*-axis end is built up, resulting in piezoelectric current which flows from the +*c*-axis end towards the -*c*-axis end. The operation mechanism of a ferroelectric-based PNG can be explained by using a volume density model. Macroscopic polarization in a ferroelectric material is generated by a polarization process, and compensation charges will be attracted at the ferroelectric material's surface to screen the polarization. When the ferroelectric material is compressed, the dipole density over the reduced ferroelectric material thickness increases. However, when the ferroelectric material is stretched, the dipole density over the increased ferroelectric material thickness declines. The variation of dipole density will result in polarization change, which needs to be compensated by a variation of the absorbed charges. As a result, piezoelectric signals are created.

#### 2.1.3. Photovoltaic Effect and PVC

Photovoltaic effect provides a direct route for converting light energy into electricity. PVC based on p-n junction or ferroelectrics is commonly utilized to achieve photoelectric conversion, as illustrated in Figure 1(c). For a PVC with p-n junction configuration, a built-in electric field at the p-n interface is created due to majority carriers' diffusion. Upon illumination, electron transition from the valance band towards the conductive band is created by absorbing high-energy photons. Consequently, excitons are created and then divided by the built-in electric field, forming free carriers. Afterwards, free electrons move towards the n-type region and free holes transfer towards the p-type region. Therefore, photocurrent is generated. Photovoltaic effect in ferroelectrics was presented about 50 years ago when continuous photovoltaic signals along the polarization direction were observed by illuminating the ferroelectrics [25]. To explain such phenomena, several theories have been presented [26, 27]. Upon irradiation, excitons are created in ferroelectric material due to photon-induced electron transition, which is similar to semiconductors. However, the driving force for electron-hole pair separation and free carrier transport in a ferroelectric PVC may be a synergy of the domain wall, depolarized field, and interfacial electric field [15, 19].

#### 2.1.4. Thermoelectric Effect and ThENG

Thermoelectric effect was first discovered in metal materials in 1821 and then observed in semiconductors [28]. As one of the most important thermoelectric effects, Seebeck effect has the ability to employ temperature gradient to generate electricity and is usually utilized to constructed ThENGs.  1Figure 1(d) demonstrates the thermoelectric effect in p-type and n-type semiconductor-based ThENGs. When a temperature gradient is built-up across a p-semiconductor, holes in the warmer region will move towards the colder region and accumulate. Consequently, thermopotential *V*_TE_ directing from the colder side to the warmer side is created inside the p-type semiconductor. Consequently, thermoelectric current is produced under short-circuit condition and moves towards the warmer side. In terms of n-type semiconductors, *V*_TE_ is formed due to electrons' diffusion, and hence, pointing from the warmer side to the colder side.

#### 2.1.5. Triboelectric Effect and TENG

Triboelectric effect results from coupling between electrification and electrostatic induction. TENG was invented in 2012 based on triboelectric effect and became a remarkable mechanical energy harvester [29]. TENG possesses four working modes which are vertical contact-separation mode, lateral-sliding mode, single-electrode mode, and freestanding triboelectric-layer mode [30]. Triboelectric effect in a vertical contact-separation type TENG is demonstrated in Figure 1(e), where Friction layer-1 is assumed to possess a stronger tendency to generate net positive charges than Friction layer-2. When the two friction materials are in contact, surface charge transfer occurs because of triboelectric effect; namely, electrons are transferred from Friction layer-1 to Friction layer-2. Consequently, Friction layer-1 is positively charged, and Friction layer-2 is negatively charged; no output is generated because of electrostatic equilibrium (Figure 1(e), I). With the separation of friction materials, electrons will be attracted by the positive charges on the Friction layer-1 surface, resulting in triboelectric current (Figure 1(e), II). When the friction materials are fully separated, the whole system realizes an equilibrium state, and no output can be observed (Figure 1(e), III). If the friction materials are about contacting again, reverse triboelectric current is created (Figure 1(e), IV). For traditional TENGs, their electric output exhibits as an alternating signal. In 2018, Liu et al. achieved direct triboelectric signals by constructing a TENG with a Schottky-contact structure, paving the way for triboelectric effect to be better integrated in multieffect coupled NGs [31].

#### 2.1.6. Coupled Effect and Multieffect Coupled NG

In the past decade, PyENG, PNG, PVC, ThENG, and TENG have been extensively studied; however, their ability of energy harvesting is relatively low due to their monotonous operation mode. Luckily, some semiconductors and ferroelectrics exhibit multiple functions; for instance, ZnO NW materials possess pyroelectricity, photovoltaic characteristics, and piezoelectricity, which provides the feasibility of integrating multiple NGs into a single device to realize multieffect coupled NGs. A multieffect coupled NG has only one pair of output electrodes, making it a remarkable candidate for integration in complicated arrays of the electronic device. So far, a variety of multieffect coupled NGs have been developed by coupling piezoelectricity, pyroelectricity, thermoelectricity, optoelectricity, and triboelectricity in a standalone device. On the one hand, multieffect coupled NGs can be utilized to simultaneously harvest multiple energies from the ambient. On the other hand, interaction of multiple effects can promote energy harvesting and conversion by modulating charge carriers' behaviour. Detailed device design and operation mechanism of multieffect coupled NGs will be introduced in Section 3.

### 2.2. Materials

So far, promising materials for use in multieffect coupled NGs mainly include semiconductors, inorganic ferroelectrics, and ferroelectric polymers. To pursue better performance, materials with different forms have been developed, such as thin films, compact ceramics, and porous bulks.

#### 2.2.1. Semiconductors

Semiconductors have been intensively investigated because of their distinctive characteristics and widely utilized in diverse electric devices, such as PVC, ThENG, TENG, PyENG, and PNG. Some semiconductors simultaneously exhibit pyroelectricity, thermoelectricity, piezoelectricity, and optoelectricity, paving the way for them to work as functional materials in multieffect coupled NGs. The commonly utilized semiconductors for multieffect coupled NGs include ZnO, CdS, SnS, Br:SnSe, GaN, and Si materials. Among these semiconductors, ZnO NWs and CdS NWs are the most frequently used materials for constructing pyro-photovoltaic NGs. The conventional way for preparing aligned ZnO NWs and CdS NWs is the hydrothermal method, where aqueous solution containing ZnNO_3_·6H_2_O and HMTA and solution comprising Cd(NO_3_)_2_·4H_2_O, glutathione, and thiourea are usually employed as the precursors [32, 33]. Dimensions and density of ZnO NWs and CdS NWs strongly depend on reactant concentration, fabrication temperature, and heating time. Although ZnO NWs and CdS NWs show good pyro-photovoltaic response to photons in an ultraviolet region, photons with lower energy cannot be effectively harvested due to their relatively wide bandgap. A method has been worked out to solve this tricky problem, namely, constructing ZnO NW- and CdS NW-based multilayer films by combining narrow-bandgap semiconductors, for instance, P3HT, CH_3_NH_3_PbI_3_, and Si materials [34–36]. To increase the pyro-photovoltaic response, carrier density of ZnO NWs has been improved by doping Cl element [37]. Another promising candidate for pyro-photovoltaic NGs is SnS NWs, which can be prepared by magnetron sputtering technology [38]. Planar SnS thin films are commonly used as the active materials for thermo-photovoltaic NGs owing to their large light absorption coefficient and high Seebeck coefficient [32]. To enhance thermopotential, semiconductor bulks have been developed. For example, Ouyang et al. fabricated [100]-oriented Br:SnSe single crystals and Gao et al. prepared ZnO bulk [17, 39]. Since thermopotential shows a proportional relationship with the temperature gradient inside thermal materials, micropores are introduced into bulky ZnO by vaporizing embedded polystyrene microspheres to increase the built-in temperature gradient [40]. In addition, Zhang and Yang developed the feasibility of InP bulks for a thermo-photovoltaic NG [16]. Very recently, Liu et al. demonstrated Si wafers as suitable tribophotovoltaic materials [41].

#### 2.2.2. Inorganic Ferroelectrics

Inorganic ferroelectrics are the most popular materials for multieffect coupled effect NGs due to their multifunctional nature, comprising pyroelectricity, piezoelectricity, optoelectricity, and dielectricity. In comparison to semiconductors, ferroelectric materials possess higher piezoelectric constant, larger dielectric constant, and abnormal photovoltaic characteristics, showing greater potential in the area of multieffect coupled NGs. So far, ferroelectric films and ceramics have been developed for multieffect coupled NGs [42–44]. Ferroelectric lead zirconate titanate (PZT) materials have drawn considerable attention owing to their large piezoelectric constant, high Curie point, and ease of polarization, and PZT films for pyro-piezoelectric NG were prepared through spin-coating technology [42]. The pyroelectric coefficient and piezoelectric constant of the films were 50 nC cm^−2^ K^−1^ and 140 pC N^−1^, respectively. Additionally, PZT ceramics have been successfully applied as the piezoelectric, pyroelectric, and photovoltaic materials for a piezo-tribo-pyro-photovoltaic NG by Zhang et al. [45]. Despite so much superiority, the toxicity of the PZT materials obstructs their further development. Recently, numerous investigations have been focused on developing eco-friendly barium titanate (BTO) and bismuth ferrite (BFO) ferroelectrics for multieffect coupled NGs. For example, Ma et al. prepared cylindrical BTO ceramics for pyro-photovoltaic NGs through a solid-state sintering method [19]. The BTO ceramics' relative density and piezoelectric coefficient were more than 95% and 370 pC N^−1^, respectively. Although the BTO ceramics have a wide bandgap of about 3.3 eV, the sintering-induced oxygen vacancy can provide intermediate energy levels, consequently extending the BTO samples' photoresponse range towards a longer wavelength. Afterwards, rectangular BTO ceramics were developed for piezo-pyro-photovoltaic NGs [15]. In addition, small-size (diameter of 10 mm) cylindrical BTO were prepared for pyro-piezoelectric NGs [46]. In comparison to BTO ceramics, ferroelectric BFO ceramics are more suitable for harvesting light energy because of their narrower bandgap. Qi et al. fabricated relatively compact BFO ceramics for a pyro-photovoltaic NG and a thermo-photovoltaic NG [44].

#### 2.2.3. Ferroelectric Polymers

Ferroelectric polymers have been intensively studied because of their softness, light weight, and excellent chemical stability. Frequently used ferroelectric polymers for multieffect coupled NGs are polyvinylidene fluoride (PVDF) and PVDF-based copolymers. PVDF is a semicrystalline material, which consists of -[CH_2_-CF_2_]- monomer units. Its pyroelectricity was revealed by Kawai in 1969 [47]. Two years later, Bergman et al. reported its pyroelectricity and ferroelectricity for the first time [48]. Generally, PVDF polymers can be classified into four major polymorphs (*α*-, *β*-, *γ*-, and *δ*-phase PVDF) according to their molecular chain conformations [49]. The PVDF *β*-phase is orthorhombic, where hydrogen (H) atoms and fluorine (F) atoms are on opposite sides of its carbon backbone forming all-trans conformation. Plenty of dipoles with the same orientation lie perpendicular to its carbon backbone because of the strong electronegativity of F atoms and electropositivity of H atoms, resulting in large spontaneous polarization and high dielectric constant. Consequently, *β*-phase PVDF exhibits better piezoelectric and pyroelectric properties in comparison to other polymorphs and is widely used for piezo-pyroelectric NGs. Owing to strong electronegativity, *β*-phase PVDF exhibits charge-accepting characteristics when it works as a friction material, and its high dielectric constant can lead to large triboelectric charge density. A variety of ways have been developed to fabricate *β*-crystal PVDF, for instance, the stretching method, melt crystallization under high pressure, rapid cooling, and poling under a high electric field [50, 51]. Electrospinning is a powerful method for fabricating *β*-phase PVDF nanofiber membranes [52]. Precursor solution is usually obtained through dissolving PVDF powders in a mixed solvent containing acetone and N,N-dimethylformamide. During electrospinning, spun nanofibers can be poled and drawn in situ, promoting the formation of the ferroelectric *β*-crystal. The most popular PVDF-based copolymers for multieffect coupled NGs are achieved by partially replacing VDF units with trifluoroethylene (TrFE). Introduction of TrFE transforms PVDF chains into all-trans conformation, promoting the growth of *β*-phase crystal. The P(VDF-TrFE) material's characteristics are determined by the relative content of VDF and TrFE. Compared to pure PVDF, copolymers containing about 20-30 mol% of TrFE exhibit higher remnant polarization. Flat P(VDF-TrFE) membranes and fibers have been prepared using a spin-coating method and electrospinning approach, respectively. In addition, to obtain enhanced piezoelectricity, Chen et al. prepared a freestanding P(VDF-TrFE) nanowire array by employing a nanoimprinting technique [53]. Another frequently utilized PVDF-copolymer material for piezo-triboelectric NGs is realized by adding hexafluoropropylene (HFP) into the PVDF matrix [54].

#### 2.2.4. Others

Despite the high stretchability of ferroelectric polymers, their relatively low spontaneous polarization remains the main obstacle for their development. Various composites on the basis of polymer matrix emerge as alternative materials for ferroelectric polymer. Frequently reported polymer matrix materials include PVDF-based polymers and PDMS polymers. Ferroelectric nanomaterials and semiconducting nanoarchitectures are usually employed as the fillers. The composites' properties strongly depend on their thickness, composition, microstructure, surface morphologies, nature of the matrix, and attributes of the fillers. Composite films with a thickness of about 100 *μ*m show attractive flexibility. Commonly used ferroelectric polymer composites for piezo-triboelectric NGs are BTO/PDMS composite films, which can be obtained by drop-casting the mixture of BTO nanoparticles and PDMS [55]. To obtain uniform films, the mixture needs to be thoroughly stirred by using a mechanical agitator or a magnetic stirrer. Addition of ~20 wt% BTO nanoparticles can lead to high remnant polarization (150 *μ*C cm^−2^) and large dielectric constant, which significantly promotes their piezoelectricity as well as triboelectricity [56]. Besides BTO nanoparticles, some other lead-free ferroelectric fillers have been utilized to prepare composites, for instance, Ba_0.7_Ca_0.3_TiO_3_-BaSn_0.12_Ti_0.88_O_3_ nanopowders and Bi_0.5_Na_0.5_TiO_3_-SrTiO_3_ nanofibers [57]. Ferroelectric polymer composites exhibit various advantages; however, a very high electric field (120-200 kV cm^−1^) and a long polarization process (10-12 h) are required to realize strong polarization. Kim et al. fabricated ZnO/PDMS composites for piezo-triboelectric NGs by using ZnO nanoflowers as the fillers [58]. For the ZnO/PDMS composites, 0.6-4.8 wt% ZnO nanoflowers could evenly disperse in PDMS, resulting in promoted surface roughness and strengthened polarization. Consequently, both triboelectricity and piezoelectricity were improved. However, excess ZnO nanoflowers (6.4-9.6 wt%) reduced the consistent orientation of electric dipoles due to agglomeration, deteriorating the piezoelectricity of the films. Considering the poor conductivity of ZnO and PDMS, MWCNT was introduced into the ZnO/PDMS composites, offering a path for charge transfer.

### 2.3. Timeline of Rapid Developments

**Figure 2:**
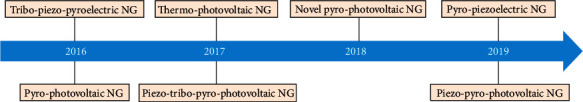
Timeline of rapid developments in multieffect coupled NGs.

In 2006, Wang and Song presented NGs, opening a new era for electronic devices [5]. Afterwards, more and more researchers devoted to developing NGs for harvesting light, thermal, chemical, and mechanical energies. As aforementioned, some materials exhibit multifunctional characteristics (triboelectricity, piezoelectricity, pyroelectricity, and optoelectricity), paving the way for the advent of multieffect coupled NGs. Multieffect coupled NGs entered a rapid development period from the year 2016 (Figure 2) when Wang et al. constructed a tribo-piezo-pyroelectric NG by using ferroelectric PVDF [59]. In the same year, Zhang and his coworkers designed a vertical-structured pyro-photovoltaic NG by constructing a P3HT/ZnO heterojunction [34]. In 2017, a thermo-photovoltaic NG on the basis of InP/ZnO heterojunction was designed [16]. At a similar time, a piezo-tribo-pyro-photovoltaic NG based on ferroelectric PZT ceramics, nylon, and FEP materials was developed [45]. The next year, pyro-photovoltaic NGs with an ITO/BTO/ITO planar structure were proposed and rapidly developed [43]. In 2019, a pyro-piezoelectric NG with a vertical-structured ITO/BTO/ITO configuration was achieved [46].

## 3. Device Design

Device configuration acts as a vital factor to determine multieffect coupled NGs' performance. Extensive researches have devoted to device design for multieffect coupled NGs. At present, sandwich layer configuration, planar structure, and heterojunction junction architecture have been constructed.

### 3.1. Sandwich Layer Configuration

**Figure 3:**
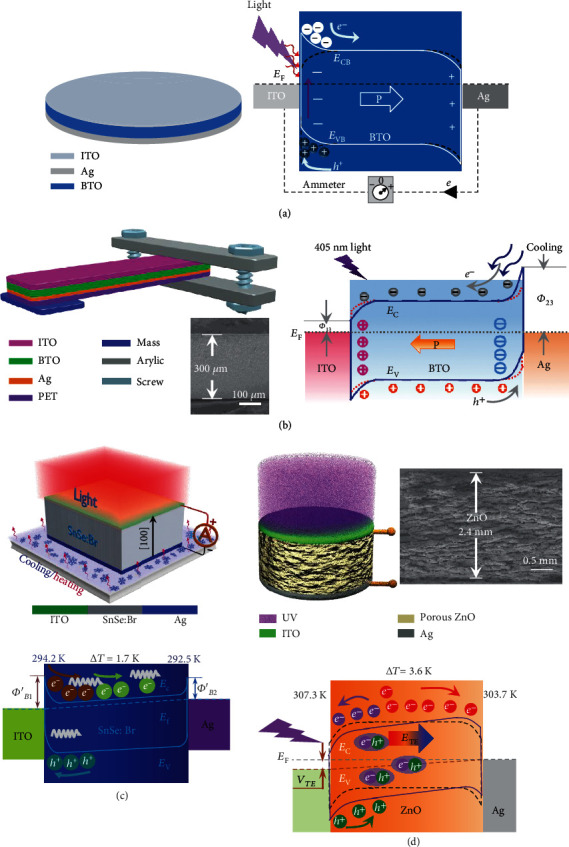
Multieffect coupled NGs with sandwich layer configuration. (a) Pyro-photovoltaic NG on the basis of sandwich-structured ITO/BTO/Ag [43]. Copyright 2018, Elsevier. (b) Piezo-pyro-photovoltaic NG with ITO/BTO/Ag sandwich configuration [15]. Copyright 2019, Royal Society of Chemistry. (c) Thermo-photovoltaic NG on the basis of ITO/Br:SnSe/Ag sandwich architecture [17]. Copyright 2019, Elsevier. (d) Thermo-photovoltaic NG based on sandwich-structured ITO/ZnO/Ag [40]. Copyright 2019, Wiley.

Multieffect coupled NGs with sandwich layer configuration are composed of functional materials embedded in two electrodes.  Figure 3(a) shows a representative sandwich-structured pyro-photovoltaic NG which consists of a ferroelectric BTO layer, an ITO layer, and an Ag film [43]. The BTO layer acts as the photovoltaic and pyroelectric materials and has a typical thickness of 0.3 mm. Schottky contact is formed at the ITO/BTO interface, leading to a built-in electric field. Upon irradiation, photons are absorbed by the BTO layer. On the one hand, electrons in the valence band can be pumped into the conductive band by photons, forming free excitons. And then, the excitons are divided by the built-in electric field and depolarized field, leading to photovoltaic signals. On the other hand, the absorbed photons can interact with the lattice of BTO and finally heat the device. Consequently, average polarization in the BTO material reduces, leading to pyroelectric outputs. Because of the same polarity of the photovoltaic signals and the pyroelectric signals, the device's performance is promoted through pyro-photovoltaic effect.  Figure 3(b) demonstrates a pyro-piezo-photovoltaic NG with sandwich layer architecture, where a BTO ceramic disk with dimensions of 38 × 3.8 × 0.3 mm^3^ works as the functional material; ITO and Ag films on its surfaces serve as the electrodes [15]. The device can be utilized to harvest light and vibration energies. Notably, vibration-induced air flow can effectively reduce the device temperature, enhancing the depolarization field and built-in electric field inside BTO. Consequently, photogenerated carrier separation and transport are improved, leading to better output performance. Ouyang et al. fabricated a thermo-photovoltaic NG with ITO/Br:SnSe/Ag sandwiched architecture, as sketched in Figure 3(c) [17]. In the device, a depletion region is formed adjacent to the ITO/Br:SnSe interface and works as the driving force for photogenerated carrier separation and transport. Upon irradiation, photocurrent towards the Ag side is created. Additionally, cooling the Ag side will lead to thermoelectric current which has the same polarity as the photocurrent. As a consequence, the thermo-photovoltaic NG exhibits better output due to the synergy of thermoelectric effect and photovoltaic effect.  Figure 3(d) shows another sandwich-structured thermo-photovoltaic NG which is constructed on the basis of porous ZnO materials [40]. ITO and Ag layers serve as electrodes, forming Ohmic contact with ZnO. The device's working principle is demonstrated by using an energy band diagram. Under illumination, a thermopotential is created across the ZnO material due to light-induced temperature gradient. In the meantime, photocurrent can be created owing to photovoltaic effect. The total current output is a consequence of thermo-photovoltaic effect. Besides rigid devices, flexible multieffect coupled NGs with a sandwiched structure have been designed. For instance, a flexible pyro-piezoelectric NG which consists of a P(VDF-TrFE) layer embedded between PDMS-CNT and graphene electrodes was constructed [56]. When the device is released and heated, the P(VDF-TrFE) film' average spontaneous polarization can be reduced, resulting in piezo-pyroelectric signals.

### 3.2. Planar Structure

**Figure 4:**
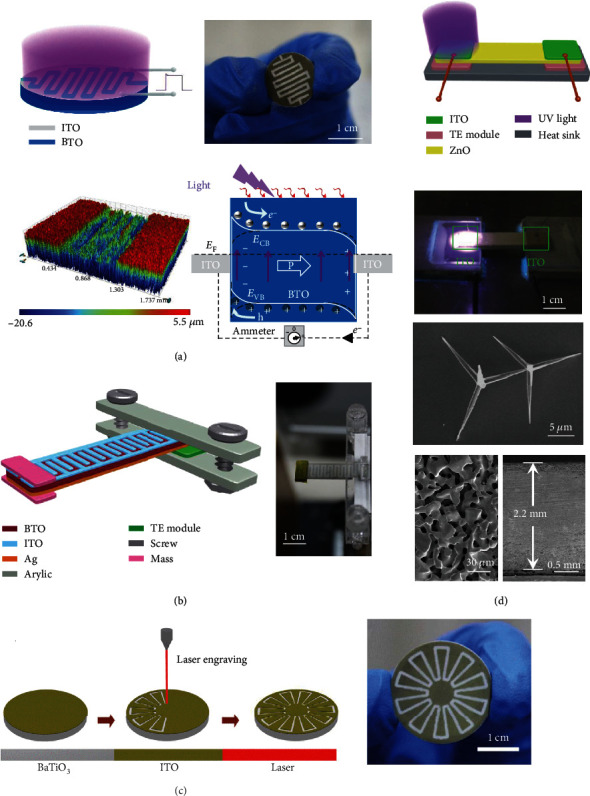
Multieffect coupled NGs with planar structure. (a) Pyro-photovoltaic NG on the basis of ITO/BTO/ITO planar configuration [43]. Copyright 2018, Elsevier. (b) Piezo-pyro-photovoltaic NG with planar-structured ITO/BTO/ITO [60]. Copyright 2019, Wiley. (c) Pyro-photovoltaic NG on the basis of ITO/BTO/ITO radially polarized planar structure [61]. Copyright 2018, Elsevier. (d) Thermo-photovoltaic NG with ITO/ZnO/ITO planar configuration [39]. Copyright 2020, Wiley.

Devices with a planar structure are mainly composed of two parts, which are a layer of functional material and coplanar electrodes. The coplanar electrodes can be realized by laser etching technology or a mask process.  Figure 4(a) demonstrates a representative planar-structured pyro-photovoltaic NG. The NG consists of a BTO ceramic disk and ITO interdigital electrodes [43]. Photons absorbed by the BTO layer can pump electrons into the conductive band, leading to photocurrent. Simultaneously, interaction between the absorbed photons and the lattice increases the temperature of the BTO, leading to pyroelectric current. Due to the same polarity of the photocurrent and pyroelectric current, the output electric signals are enhanced by pyroelectric-photovoltaic effect. It is worth noting that the incident light can directly illuminate the exposed parts of the BTO layer, enhancing the interaction between the photons and the BTO material. Consequently, in comparison to sandwich-structured devices, better output can be achieved by planar-structured pyro-photovoltaic NGs. The planar structure also provides the feasibility for multieffect coupled NGs to improve their piezoelectric response.  Figure 4(b) illustrates a piezo-pyro-photovoltaic NG with a planar structure [60]. The functional component is a BTO ceramic disk with dimensions of 38 × 3.8 × 0.6 mm^3^. A pair of interdigital electrodes is positioned on the BTO disk's surface, allowing the device to employ piezoelectric *d*_33_ mode to generate higher piezoelectric response. Another planar-structured pyro-photovoltaic NG is shown in Figure 4(c), where the radially polarized ITO electrodes on the BTO disk are realized by laser etching technology [61]. It is found that the pyro-photovoltaic output strongly depends on the quantity of electrodes.  Figure 4(d) displays an example of a planar-structured thermo-photovoltaic NG which is constructed by utilizing a ZnO bulk [39]. Coplanar ITO electrodes are on both ends of the ZnO bulks. When one of the ITO electrodes is irradiated, temperature gradient is built up across the ZnO sample. The coplanar design effectively increases the electrode gap and results in a higher temperature gradient. Consequently, the thermo-photovoltaic output can be improved.

### 3.3. Heterojunction Architecture

**Figure 5:**
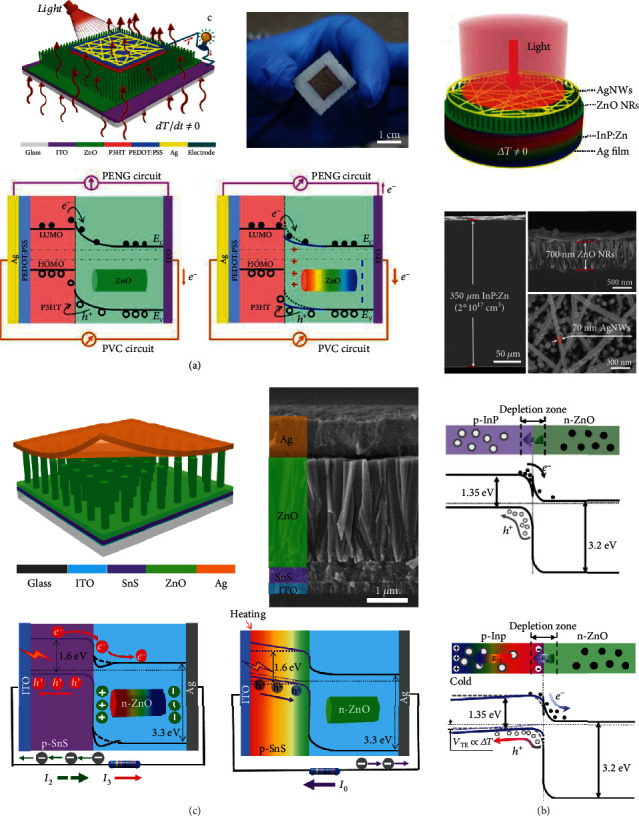
Multieffect coupled NGs with heterojunction architecture. (a) Pyro-photovoltaic NG with P3HT/ZnO heterojunction [34]. (b) Thermo-photovoltaic NG based on InP/ZnO heterojunction [16]. Copyright 2017, Wiley. (c) Pyro-photovoltaic and thermo-photovoltaic NGs with SnS/ZnO heterojunction [32]. Copyright 2018, Elsevier.

Heterojunction is commonly utilized in PVCs, and n-type ZnO NWs are usually employed to construct p-n junction. As aforementioned, ZnO NWs exhibit remarkable thermoelectric and pyroelectric properties, paving the way for constructing heterojunction-based multieffect coupled NGs.  Figure 5(a) illustrates a pyro-photovoltaic NG with P3HT/ZnO NW heterojunction, where the ZnO NW film has a thickness of ~3 *μ*m [34]. An Ag/PEDOT:PSS composite film and an ITO layer are employed as electrodes. This device can be viewed as an integration of a PyENG and a PVC. Upon illumination, photocurrent flowing towards the ITO film can be created because of photoexcited carriers' movement. During this process, the light-induced heating is negligible due to the low light intensity (77 Lux to 2340 Lux); consequently, no output is generated in the PyENG. Once a cooling process is applied on the ITO side during illumination, the total polarization of the ZnO NWs increases, and more positive charges move towards the P3HT/ZnO interface, lowing ZnO material's conductive band. And therefore, it is easier for the electrons to leap over the barrier of the P3HT/ZnO interface, improving the performance of PVC. In addition, more compensation charges are attracted to the electrodes due to enhanced polarization, which leads to pyroelectric current. Finally, the device's output signals are promoted because of the same flowing direction of the photocurrent and pyroelectric current. A thermo-photovoltaic NG with InP/ZnO heterojunction is shown in Figure 5(b) [16]. The device operates as a p-n junction PVC when it is illuminated. However, some photogenerated electrons are trapped at the InP/ZnO interface because of band bending and band offset, resulting in relatively low photovoltaic current. When a temperature gradient is created inside the InP wafer by cooling the device, holes move to the colder side and accumulate, leaving behind negative charges near the InP/ZnO interface. Consequently, the conductive band of the InP is elevated and the trapped electrons can escape from the InP/ZnO interface into the conductive band of ZnO, promoting the output of the device. Ouyang et al. designed a function-switchable multieffect coupled NG based on SnS/ZnO heterojunction, as sketched in Figure 5(c) [32]. The length of the ZnO NWs is around 2 *μ*m. ITO and Ag layers are served as electrodes. The combination of thermoelectric SnS material and pyroelectric ZnO material makes it possible for the device to work as a pyro-photovoltaic NG or a thermo-photovoltaic NG by modulating the wavelength of incident light. Under visible light irradiation, photons can be absorbed by the SnS material, resulting in photocurrent. Additionally, a part of photons can go through the SnS film and heat the ZnO NWs, resulting in pyroelectric current. Since the photocurrent has the same polarity as the pyroelectric current, the total output current is promoted due to pyro-photovoltaic effect. Upon ultraviolet light irradiation, all the photons are absorbed by the SnS film and thermo-photovoltaic current is produced and flows towards the ITO electrode. Except for the aforementioned devices, a pyro-photovoltaic NG with Si/CdS heterojunction and a thermo-photovoltaic NG with CIGS/CdS heterojunction have also been constructed [33, 62].

### 3.4. Others

**Figure 6:**
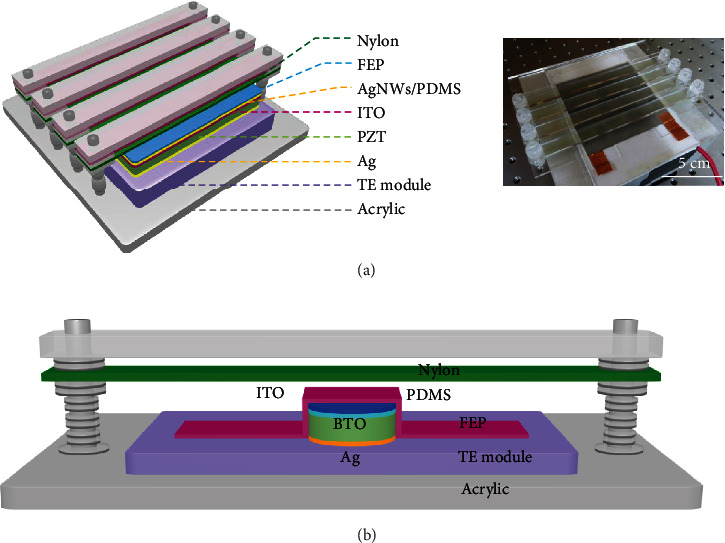
Multieffect coupled NGs with hybrid architectures. (a) Piezo-tribo-pyro-photovoltaic NG with PZT-FEP/nylon hybrid structure [45]. Copyright 2017, Wiley. (b) Piezo-tribo-pyro-photovoltaic NG with BTO-FEP/nylon hybrid configuration [63]. Copyright 2018, Wiley.

The diversity of ambient energies presents a challenge in device configuration. Seeking practical and novel device architecture is always a goal, and a lot of researchers have devoted to developing state-of-the-art devices. For instance, Zhang et al. devised a piezo-tribo-pyro-photovoltaic NG by integrating a TENG and a piezo-pyro-photovoltaic NG in one device (Figure 6(a)) [45]. The NG is mainly composed of a PZT disk as piezo-pyro-photovoltaic material and a nylon layer as well as a FEP layer as rubbing layers. An ITO/Ag NW layer as well as an Ag film on the surfaces of PZT is employed as electrodes. The FEP layer is attached on the ITO/Ag NW electrode, and the nylon films are fixed above the FEP. The nylon film can vibrate when air flows through, rubbing with the FEP and applying a strain on the PZT disk. Consequently, piezo-triboelectric signals are created. Light and thermal energies can be effectively scavenged by employing pyro-photovoltaic effect of the PZT material. Figure 6(b) illustrates another piezo-tribo-pyro-photovoltaic NG which is constructed by using ferroelectric BTO, nylon, and FEP materials [63]. Utilization of the nontoxic BTO material makes the device suitable for daily application. In recent years, direct-current TENGs have attracted intense interest since it provides a novel direction for coscavenging of light and mechanical energies. Liu et al. fabricated a tribo-photovoltaic NG with stainless steel/Si/Al configuration [41]. When friction and irradiation are simultaneously applied, tribo-tunneling current is created and flows towards the Al side. Meanwhile, photoexcited excitons are produced, subsequently separated under the force of rubbing-induced interfacial electric field and Fermi level difference-induced built-in electric field, forming free carriers. The obtained carriers transport and are finally collected by the electrodes, resulting in photocurrent. The total output electric signals are enhanced because of the improved carrier lifetime and the same polarity of tribo-tunneling current and photocurrent.

## 4. Output Characteristics

Output performance of multieffect coupled NGs exhibits different characteristics depending on functional materials, device structure, working principle, and operation conditions. Output current as well as output voltage is a vital parameter to assess the device's performance.

### 4.1. Output Current

**Figure 7:**
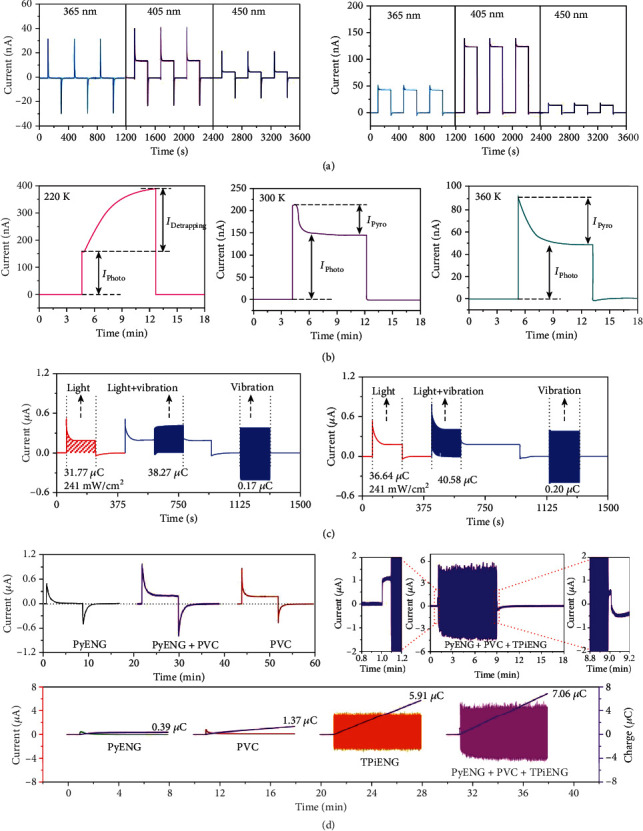
Output current of multieffect coupled NGs. (a) Current signals generated by sandwiched ITO/BTO/Ag pyro-photovoltaic NG as well as planar ITO/BTO/ITO pyro-photovoltaic NG [43]. Copyright 2018, Elsevier. (b) Temperature-dependent current produced by a planar ITO/BTO/ITO pyro-photovoltaic NG [64]. Copyright 2019, Elsevier. (c) Current signals generated by a piezo-pyro-photovoltaic NG with ITO/BTO/Ag configuration [15]. Copyright 2019, Royal Society of Chemistry. (d) Current delivered by a piezo-tribo-pyro-photovoltaic NG with PZT-FEP/nylon hybrid configuration [45]. Copyright 2017, Wiley.

Figure 7(a) exhibits representative output current signals of BTO-based pyro-photovoltaic NGs under illumination [43]. The top time-dependent current patterns are related to an ITO/BTO/Ag NG with sandwich configuration, and the bottom ones are from an ITO/BTO/ITO NG with planar architecture. All the current-time curves exhibit as representative pyro-photoelectric signals which consist of sharp peaks and stable platforms. When the devices are illuminated, photocurrent is created due to photovoltaic effect of the BTO material. In the meantime, the temperature of the BTO layer rapidly increases because of photothermal effect, leading to instantaneous pyroelectric current. As aforementioned, the photocurrent and the pyroelectric current have the same polarity; consequently, a sharp pyro-photovoltaic current peak is observed. And then, the temperature of the device gradually becomes steady with the illumination time prolonging; consequently, the pyroelectric current reduces and finally vanishes, leaving behind stable photocurrent (platform current). In comparison to sandwich-structured NGs, NGs with planar architecture can deliver higher pyro-photovoltaic current. Explanations for such an interesting phenomenon may be that more photons can reach and be absorbed by the BTO ceramics in planar-structured NGs, enhancing the coupling of pyroelectricity, optoelectricity, and ferroelectricity of the BTO material. Temperature-dependent current of planar-structured ITO/BTO/ITO pyro-photovoltaic NGs has been studied by Ma and Yang, as illustrated in Figure 7(b) [64]. Current-time curves obtained at 220 K, 300 K, and 360 K exhibit various shapes, suggesting that different mechanisms are involved in the device. When the ambient temperature is low (220 K), the current rapidly increases to a small value and then increases slowly to a higher value (393.1 nA) under 405 nm light illumination. As is reported, free electrons are easier to be trapped by an impurity-induced shallow trap level. The fast and the slow current enhancement is ascribed to the band-band excitation and shallow trap level-related excitation, respectively. As the temperature increases, less electrons are trapped; consequently, the slowly increasing part of the current becomes lower at 300 K and finally vanishes at 360 K. Figure 7(c) exhibits current signals of a BTO-based piezo-pyro-photovoltaic NG under different working conditions [15]. When the device is illuminated or vibrated, typical pyro-photovoltaic current (0.55 *μ*A) or piezoelectric current (0.384 *μ*A) is observed. By simultaneously applying light and vibration, the peak value as well as the transferred charge is significantly increased owing to piezo-pyro-photovoltaic effect, and analogous behaviour is observed even if changing the sequence of the applied light and vibration stimuli. Figure 7(d) shows current signals of a piezo-tribo-pyro-photovoltaic NG [45]. The peak current signals of the device under PyENG and PVC modes are 0.48 *μ*A and 0.17 *μ*A, respectively, which can be improved to about 1 *μ*A under a PyENG+PVC operation mode. Moreover, the addition of TPiENG can further promote the device's current as well as transferred charge.

### 4.2. Output Voltage

**Figure 8:**
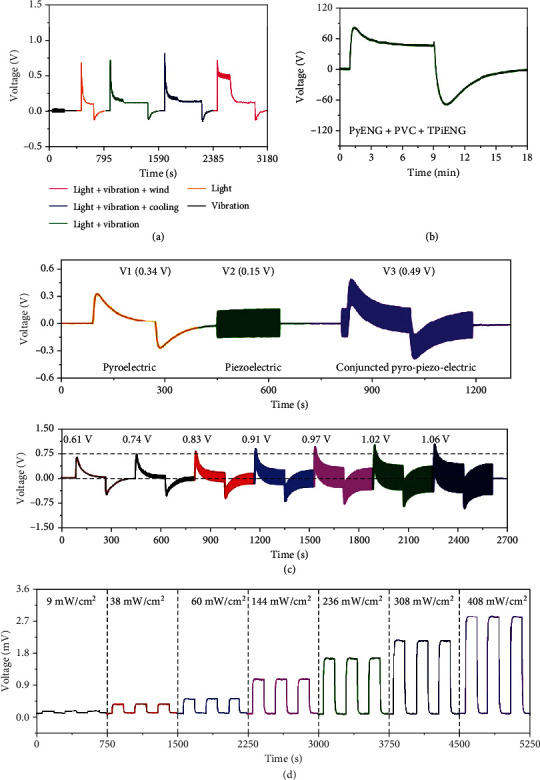
Output voltage of multieffect coupled NGs. (a) Voltage signals generated by a pyro-piezo-photovoltaic NG with ITO/BTO/Ag configuration [15]. Copyright 2019, Royal Society of Chemistry. (b) Voltage of a piezo-tribo-pyro-photovoltaic NG with PZT-FEP/nylon hybrid configuration [45]. Copyright 2017, Wiley. (c) Voltage produced by a pyro-piezoelectric NG on the basis of Ag/BTO/Ag configuration [46]. Copyright 2019, Wiley. (d) Output voltage of a thermo-photovoltaic NG with ITO/ZnO/Ag configuration [40]. Copyright 2019, Wiley.

Figure 8(a) illustrates the voltage-time curves generated by a piezo-pyro-photovoltaic NG with an ITO/BTO/Ag structure [15]. The voltage exhibits a sharp peak of 0.67 V followed by a platform of 0.1 V under irradiation (405 nm). The peak value is ascribed to the pyro-photovoltaic effect, and the stable platform voltage resulted from photovoltaic effect. The peak voltage can be significantly improved by pyro-piezo-photovoltaic effect under synergy of light, vibration, and cooling. Additionally, the platform voltage is more sensitive to the device temperature and can be greatly improved by vibration- and wind-induced cooling effect. Figure 8(b) illustrates the output voltage of a piezo-tribo-pyro-photovoltaic NG [45]. The voltage signals strongly relied on the device's operation mode. The maximum platform voltage of 46 V can be obtained when it acts as a PyENG+PVC+TPiENG. Voltage signals of a stainless steel/Si/Al tribo-photovoltaic NG have been investigated by Liu et al. [41]. The output voltage induced by tribo-tunneling transport is about 0.2 V, which can be increased to 0.35 V by illuminating the sliding contact region. Song et al. investigated voltage signals of an Ag/BTO/Ag pyro-piezoelectric NG (Figure 8(c)) [46]. Pyroelectricity-induced and piezoelectricity-induced peak voltage signals are 0.34 V and 0.15 V, respectively. When both heating and pressing are presented, the peak voltage can be improved to 0.49 V. Furthermore, the pyro-piezoelectric voltage shows a proportional relationship with the applied pressure or temperature gradient increasing. Figure 8(d) shows the time-dependent thermo-photovoltaic voltage of a sandwich-structured ITO/ZnO/Ag device under irradiation, exhibiting excellent reliability and good linear relationship with light intensity [40].

## 5. Applications

With the electric devices' working environment changes, light, thermal, and mechanical energies can be resources to powering diverse electronics. By coupling pyroelectric effect, piezoelectric effect, thermoelectric effect, and triboelectric effect in a single device, multieffect coupled NGs possess the superiority of lightweight, versatility, and portability, showing powerful ability for energy scavenging as well as self-powered sensing.

### 5.1. Enhanced Light Energy Harvesters

**Figure 9:**
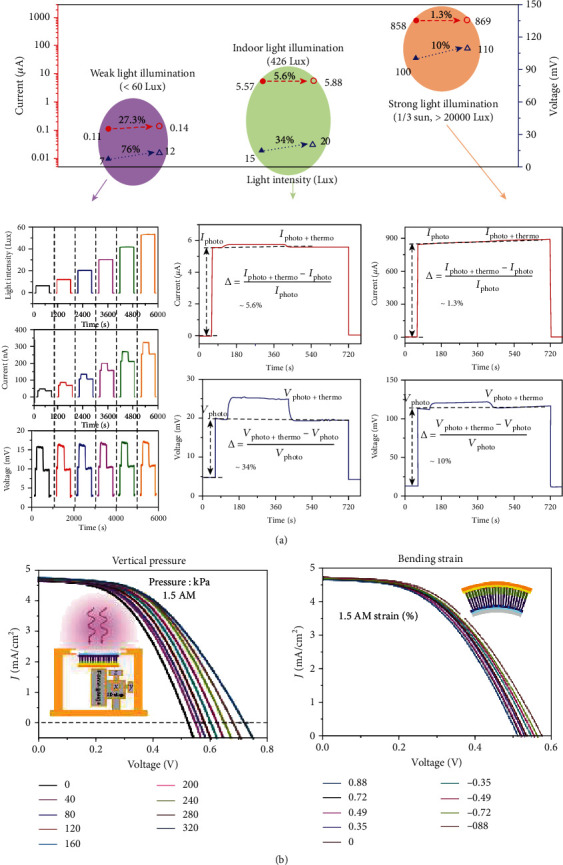
Multieffect coupled NGs utilized as enhanced light harvesters. (a) Performance of a thermoelectricity-enhanced light harvester [16]. Copyright 2017, Wiley. (b) Promoted performance of a piezoelectricity-enhanced light harvester, where *J*_SC_ is short-circuit current density, *V*_OC_ stands for open-circuit voltage, and *η* is conversion efficiency [65]. Copyright 2017, Wiley.

PVC is one of the best routes for converting light energy into electricity. Searching effective approaches for improving the PVCs' output is a long-term goal. Up to now, lots of effort has been committed to promote PVCs' performance, such as optimizing their manufacturing process, modulating the bandgap of photovoltaic materials, constructing tandem devices, and reducing rear surface reflection. Approaches aforementioned usually require harsh technical conditions and high costs. Coupling between photovoltaic effect and other electric effect provides a convenient way of promoting PVC's output electricity. In this regard, thermo-photovoltaic, tribo-photovoltaic, and piezo-photovoltaic effects show great potential.  Figure 9(a) demonstrates the photovoltaic output of a thermoelectricity-enhanced light harvester on the basis of InP/ZnO architecture, where a temperature gradient of 3.5°C is applied [16]. The enhancement of photocurrent and photovoltage is more evident under weaker light illumination, and the maximum enhancement ratios of photocurrent and photovoltage are 27.3% and 76%, respectively. Zhu et al. demonstrated the piezoelectricity-enhanced photovoltaic performance of a piezo-photovoltaic NG (Figure 9(b)) [65]. The device is constructed on the basis of SnS/ZnO NW heterojunction. To evaluate the influences of piezopotential, *J*‐*V* characteristics under various vertical pressures and bending strain are recorded. It is found that *J*_SC_, *V*_OC_, and *η* are increasingly proportional to the employed vertical pressure, while the fill factor (FF) is almost unchanged. *J*_SC_, *V*_OC_, and *η* are increased by 2.4%, 39%, and 37.3% by applying a 320 kPa pressure, respectively. The performance promotion is ascribed to the piezopotential-induced enhancement of the built-in electric field. Based on the same principle, *J*_SC_, *V*_OC_, and *η* can be promoted by compressing the device. By applying a -0.88% compressive strain, *J*_SC_ and *V*_OC_ can be promoted to 4.71 mA cm^−2^ and 580 mV, respectively. Meanwhile, *η* can be promoted to 1.3%. Liu et al. studied the triboelectricity-enhanced photovoltaic performance of a tribo-photovoltaic NG with a stainless steel/Si/Al structure [41]. The photocurrent is only ~1 *μ*A under illumination (90 mW cm^2^), which can be increased to about 4 *μ*A by the synergy of triboelectricity and optoelectricity; however, the photovoltage is almost invariable.

### 5.2. Self-Powered Photodetectors

**Figure 10:**
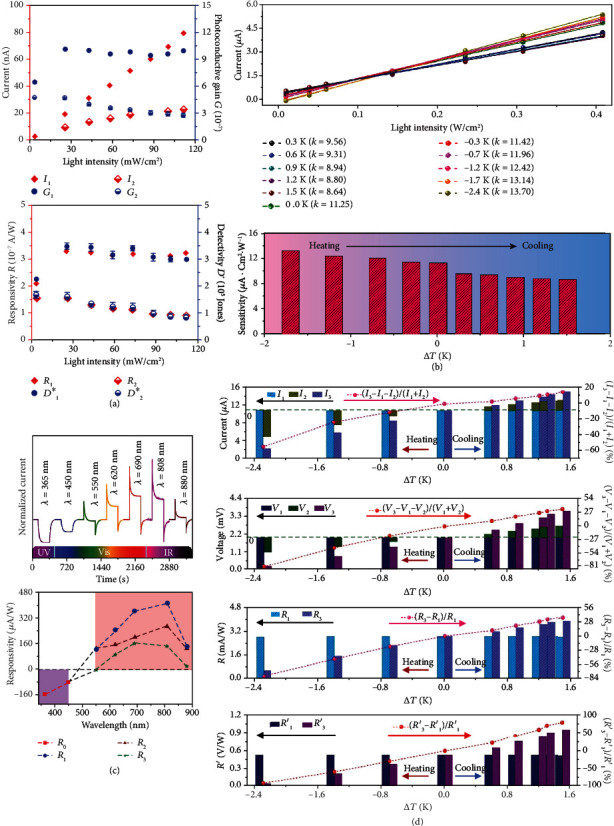
Multieffect coupled NGs for self-powered photodetectors. (a) Photodetection performance of a pyro-photovoltaic NG for monitoring 405 nm light, where *I*_1_, *G*_1_, *R*_1_, and *D*^∗^_1_ are associated with pyro-photovoltaic effect, and *I*_2_, *G*_2_, *R*_2_, and *D*^∗^_2_ are associated with photovoltaic effect [19]. Copyright 2017, Wiley. (b) Detection performance of a thermo-photovoltaic NG for sensing 365 nm light [40]. Copyright 2019, Wiley. (c) Performance of pyro-photovoltaic and thermo-photovoltaic NGs for distinguishing light wavelength [32]. Copyright 2018, Elsevier. (d) Performance of a thermo-photovoltaic NG for detecting 760 nm light [17]. Copyright 2019, Elsevier.

A self-powered photodetector is another influential application of multieffect coupled NGs, which can operate without external power supplies and meet the small-size, lightweight, and low-power-consumption demands for next-generation electronics.  Figure 10(a) exhibits the photodetection performance of a pyro-photovoltaic NG [19]. The device has an ITO/BTO/Ag sandwiched structure and can be utilized for 405 nm light detection. Both *I*_1_ and *I*_2_ linearly increase as the light intensity promotes, indicating that light intensity can be perfectly reflected by analyzing *I*_1_ and *I*_2_. Additionally, *G*_1_, *R*_1_, and *D*∗_1_ show a higher value than *G*_2_, *R*_2_, and *D*∗_2_. Qi et al. devised an ultraviolet photodetector utilizing an ITO/ZnO/Ag thermo-photovoltaic NG [20].  Figure 10(b) depicts the performance of the device when temperature gradients of -2.4 to 1.5 K are applied across the ZnO material. Under any temperature gradients, linearly increasing output current can be observed. The sensitivity of the device can be obtained by linearly fitting the light-intensity-dependent current curves. As can be seen, the sensitivity is variable, showing an inversely proportional relationship to the applied temperature gradient. By employing a -2.4 K temperature gradient, the sensitivity can be improved to 13.7 *μ*A cm^2^ W^−1^.  Figure 10(c) depicts the photodetection performance of a SnS/ZnO heterojunction-based multieffect coupled NG [32]. When stimulated by ultraviolet and visible light, the photodetector can deliver current signals with opposite polarities. For instance, the device can deliver a negative current signal due to thermo-photovoltaic effect upon 365 nm light illumination, however generate a positive pyro-photovoltaic current signal under 690 nm light irradiation. The responsivity of the photodetector exhibits a rising relationship as the light wavelength increases in the range of 365-808 nm. Furthermore, the responsivity *R*_1_ related to pyro-photovoltaic effect is higher than the pyroelectricity- and optoelectricity-induced responsivity (*R*_2_ and *R*_3_).  Figure 10(d) exhibits the performance of a 760 nm photodetector on the basis of an ITO/Br:SnSe/Ag thermo-photovoltaic NG, where *I*_1_, *V*_1_, *R*_1_, and *R*_1_′ are related to photovoltaic effect, *I*_2_ and *V*_2_ are relevant to thermal effect, and *I*_3_, *V*_3_, *R*_3_, and *R*_3_′ are related to thermo-photovoltaic effect [17]. It is found that *I*_3_, *V*_3_, *R*_1_′, and *R*_3_′ are significantly enhanced by cooling the device's Ag side, and the enhancement ratio almost linearly increases as the temperature gradient rises. By building up a 1.5 K temperature gradient, *I*_3_, *V*_3_, and *R*_3_′ can be promoted to 15.1 *μ*A, 3.67 mV, and 0.97 V W^−1^, respectively. More self-powered photodetectors on the basis of multieffect coupled NGs are summarized in Table 1 [20, 35, 43, 44, 66–70].

### 5.3. Multienergy Harvesters

**Figure 11:**
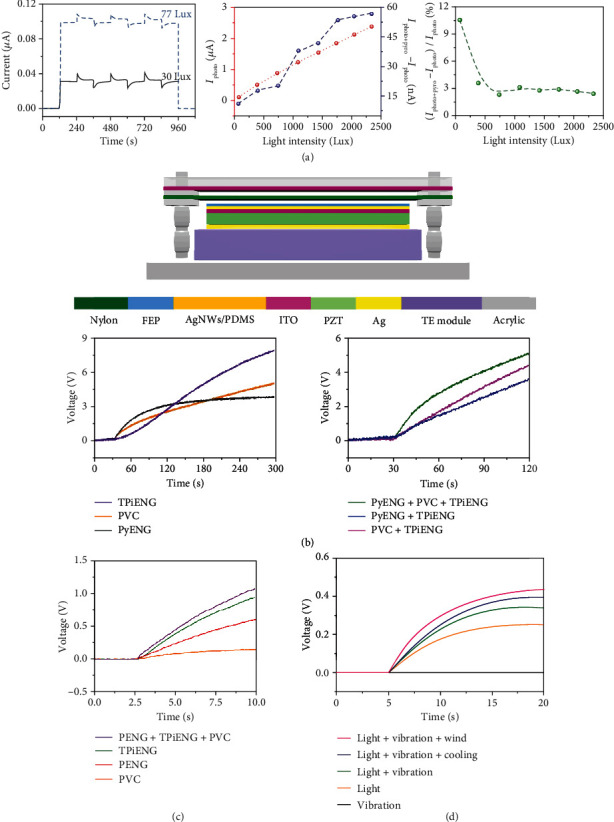
Multieffect coupled NGs for multienergy harvesters. (a) Performance of a multieffect coupled NG for harvesting light and heat [34]. Copyright 2016, American Chemical Society. (b) Multieffect coupled NG utilized to collect solar energy, heat, and wind energy [45]. Copyright 2017, Wiley. (c) Charging characteristics of a piezo-tribo-pyro-photovoltaic NG for harvesting 405 nm light, thermal, and wind energies [63]. Copyright 2018, Wiley. (d) Multieffect coupled NG for harvesting light and vibration energies [15]. Copyright 2019, Royal Society of Chemistry.

Converting ambient energy into electricity is a long-term goal. Over the past few years, lots of researchers were devoted to constructing NGs to scavenge individual light, thermal, and mechanical energies. However, the applications of these NGs are quite limited because of their single working mode. Multieffect coupled NGs exhibit strong ability of simultaneously harvesting multitype energies.  Figure 11(a) illustrates the output performance of a P3HT/ZnO heterojunction pyro-photovoltaic NG [34]. The device can be utilized to simultaneously harvest light and heat. Upon continuous illumination with light intensities of 30 Lux and 77 Lux, the device can deliver stable photovoltaic current signals of 30 nA and 100 nA, respectively. By cooling the device (peak rate of ~1.2 K/s), the current signals are increased to 71 nA (30 Lux) and 210 nA (77 Lux) due to pyro-photovoltaic effect. Furthermore, the photocurrent as well as the pyroelectric current promotes with the light intensity increasing, leading to higher pyro-photovoltaic current signals. The current enhanced ratio reaches a maximum value of 10% upon weak illumination.  Figure 11(b) depicts a piezo-tribo-pyro-photovoltaic NG which is constructed by integrating PZT, nylon, and FEP materials in a single device [45]. The device acts as a PVC, a PyENG, and a TPiENG under the individual stimulus of solar, thermal, and wind energies, respectively. A 4.7 *μ*F capacitor's voltage is measured to evaluate the device's performance. When the capacitor is charged by individual PVC, PyENG, and TPiENG, its voltage can be promoted to about 3 V after a 120 s charging process. However, when the PyENG, PVC, and TPiENG work simultaneously, the capacitor's voltage can be increased to 5 V in 120 s. Similar phenomena can be observed by using a BTO-based piezo-tribo-pyro-photovoltaic NG (Figure 11(c)) [63]. A cantilever-structured piezo-pyro-photovoltaic NG was designed for synchronously collecting light energy as well as vibration energy [15]. The core component of the device is a cuboid BTO ceramic which is sandwiched between ITO and Ag electrodes. The voltage of a 0.33 *μ*F capacitor is demonstrated in Figure 11(d). It can be found that the charging speed is significantly promoted by simultaneously harvesting light and vibration energies.

### 5.4. Self-Powered Multifunctional Sensors

**Figure 12:**
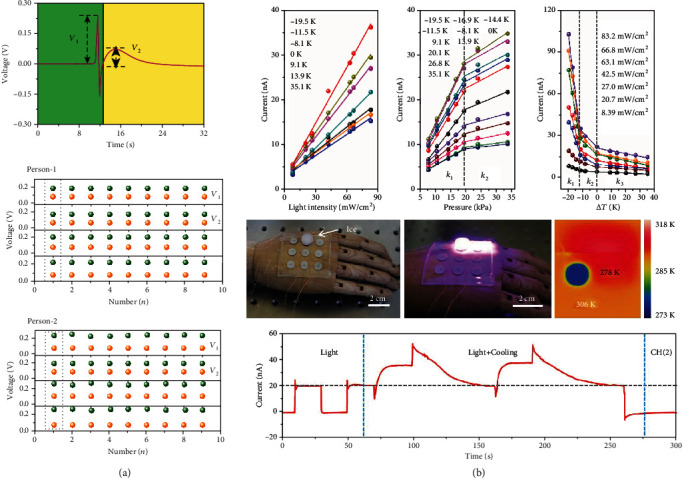
Multieffect coupled NGs utilized as multifunctional sensors. (a) Pressure-temperature detection performance of a piezo-pyroelectric NG with ITO/BTO/Ag structure [46]. Copyright 2019, Wiley. (b) Light, pressure, and temperature detection performance of a BTO-based pyro-piezo-photovoltaic NG [72]. Copyright 2020, Elsevier.

With the increasing demands for miniature sensors, self-powered multifunctional sensors which can simultaneously monitor multiple stimuli, including light, pressure, and temperature, have attracted intensive attention. Multieffect coupled NGs exhibit great potential as multifunctional sensors due to their versatility, miniaturization, fast response, and high responsivity. In recent years, flexible multifunctional sensors based on multieffect coupled NGs have been extensively investigated in the field of wearable electronics and robots [71]. For instance, to achieve temperature and pressure detection, Song et al. devised flexible pyro-piezoelectric NGs through embedding Ag/BTO/Ag devices in PDMS materials; the performance of the device is shown in Figure 12(a) [46]. Two distinct types of voltage curves can be observed when the device is touched by a finger. The instantaneous peak voltage *V*_1_ of 0.232 V mainly resulted from piezoelectric effect, and the slowly increased voltage *V*_2_ of 0.071 V is induced by pyro-piezoelectric effect. To test the device's practicality, pressures of 17.4 kPa and 18.2 kPa are applied on the device by Person-1 and Person-2, respectively. It is found that *V*_1_ of Person-2 is larger; however, *V*_2_ is lower than that of Person-1, suggesting the feasibility for the device to be utilized for synchronously detecting pressure and temperature. On the basis of an ITO/BTO/Ag piezo-pyro-photovoltaic NG, a multifunctional sensor for detecting light intensity, temperature, and pressure was constructed [72]. Figure 12(b) illustrates the device's detection performance. The pyro-photovoltaic current monotonically rises with the light intensity increases. Larger current output and higher photodetection sensitivity can be obtained at a lower temperature. When the device's temperature is reduced by 19.5 K, its photodetection sensitivity can be promoted to 0.42 nA mW^−1^ cm^2^. In terms of pressure detection, the piezoelectric current exhibits an increasing relationship with the applied pressure. The maximum pressure sensitivity of the device is 1.43 nA kPa^−1^. Furthermore, the device can be utilized as a temperature detector by analyzing temperature-dependent photocurrent. Upon continuous irradiation, a stable photocurrent is created, which can be varied with the ambient temperature. As the temperature variation increases, the photocurrent reduces. The highest temperature sensitivity of the device is reported to be -8.85 nA K^−1^. Except for ferroelectric-based devices, semiconductor-based multifunctional sensors have also been realized. For example, Gao et al. integrated photodetection and temperature sensing in a ZnO-based thermo-photovoltaic effect NG [39]. Excellent independence between thermoelectric signals and photovoltaic signals is observed in the device, which enables the device to simultaneously monitor light intensity and temperature change.

### 5.5. Self-Powered Image Sensors

**Figure 13:**
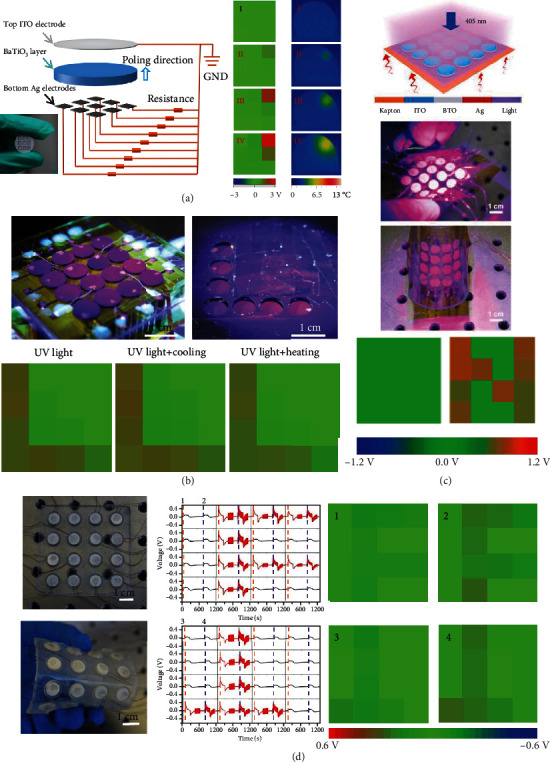
Multieffect coupled NGs for image sensors. (a) Photodetection array on the basis of ITO/BTO/Ag pyro-photovoltaic NGs [19]. Copyright 2017, Wiley. (b) Photodetection matrix based on ITO/BFO/Ag thermo-photovoltaic NGs [20]. Copyright 2018, American Chemical Society. (c) Multifunctional detector matrix on the basis of ITO/BTO/Ag pyro-piezo-photovoltaic NGs [44]. Copyright 2018, American Chemical Society. (d) Multifunctional detector array based on Ag/BTO/Ag pyro-piezoelectric NGs [46]. Copyright 2019, Wiley.

Detection for stimulus distribution is also a vital application of multieffect coupled NGs, such as self-powered photodetector arrays, self-powered pressure image sensors, and self-powered temperature sensor arrays. Ma et al. developed a visible-light photodetector array utilizing pyro-photovoltaic NGs [19]. The device is composed of a poled BTO ceramic layer, an ITO transparent electrode, and a 3 × 3 Ag electrode matrix (Figure 13(a)). Each Ag electrode has the dimensions of 2.5 × 2.5 mm^2^. Under short-time illumination, the photodetector array exhibits high resolution (Figure 13(a), I). However, the device suffers a relatively low resolution under a long-term illumination process due to the spread of light-induced heat (Figure 13(a), II, III). Figure 13(b) illustrates a flexible self-powered photodetector matrix, which consists of a 4 × 4 ITO/BTO/Ag pyro-photovoltaic NG matrix [73]. The detector units are integrated on an Ag film covered kapton. In the system, the kapton is utilized as interconnections of the rigid sensing units, improving the device's flexibility. The device can be easily bent and twisted to adapt to diverse testing condition. Furthermore, the device shows high resolution because of the relative independence among the sensing units. A recent application of thermo-photovoltaic NG as a self-powered image sensor is the BFO-based self-powered photodetector array for monitoring 365 nm ultraviolet light distribution [20]. The device consists of a 4 × 4 detector matrix with detection units integrated on an Ag film covered kapton (Figure 13(c)). Each unit has a sandwiched structure with a BFO ceramic embedded between ITO and Ag electrodes. The detector array is able to precisely monitor the intensity and location of incident light. Additionally, the device's responsivity can be greatly improved by applying a cooling process. Figure 13(d) exhibits a multifunctional image sensor relying on pyro-piezoelectric NGs [46]. The sensor is composed of a 4 × 4 sensor element array. Each unit consists of a cylindrical BTO disk with diameter of 1 cm and Ag electrodes on both sides. The Ag/BTO/Ag elements are embedded in the PDMS matrix to perfect the device's flexibility and integrity. By detecting pyroelectric voltage, the device can be employed to monitor temperature variation. For instance, heating an F-shaped area and an inverted T-shaped area of the device, corresponding patterns can be observed due to pyroelectric effect. Such patterns become more evident by pressing the stimulated areas due to piezo-pyroelectric effect.

### 5.6. Others

**Figure 14:**
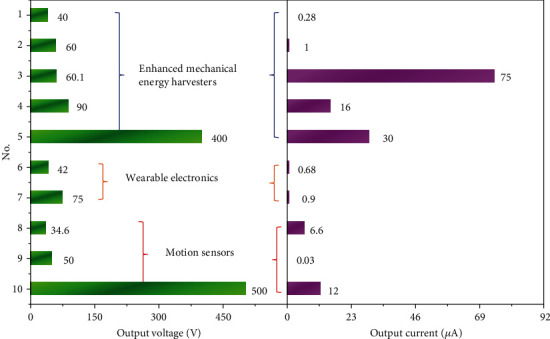
Extended applications of multieffect coupled NG. No. 1: Yang and Daoud [75]; No. 2: Shi et al. [55]; No. 3: Chowdhury et al. [76]; No. 4: Bu et al. [77]; No. 5: Kim et al. [58]; No. 6: Wu et al. [54]; No. 7: Yu et al. [78]; No. 8: Zhu et al. [79]; No. 9: Sahatiya et al. [80]; No. 10: Guo et al. [52].

In the recent years, Internet of Everything (IoE) becomes a hotspot. In the future, a complicated matrix of generators and sensors will be employed in daily life. As multifunctional electronics, multieffect coupled NGs show great potential in paving the way for IoE. Apart from the fields aforementioned, multieffect coupled NGs have been successfully employed in many other application areas and exhibit excellent performance, as illustrated in Figure 14 [59–65, 74].

## 6. Conclusions and Prospects

Multieffect coupled NGs are emerging as the coupling of thermoelectricity, pyroelectricity, triboelectricity, optoelectricity, piezoelectricity, semiconductors, and ferroelectrics, which will definitely prove to be one of the most helpful electronics in the coming IoE era because of their matchless versatility, high reliability, and good sustainability. Interaction between thermoelectric, pyroelectric, triboelectric, optoelectric, and piezoelectric effects plays an important role in controlling carriers' production, separation, movement, and recombination process and finally influencing energy scavenging and conversion. Synergy of various effects makes the multieffect coupled NGs suitable for scavenging whatever energies are available. Stimuli-dependent output allows the multieffect coupled NGs to be used as self-powered sensors. High integration and unmatched versatility provide the feasibility of the multieffect coupled NGs being integrated into diverse electric arrays even skins. Significant advances of multieffect coupled NGs have been obtained; however, several issues need to be considered, including the following: (1) Further revealing the interaction between various effects is essential. Better comprehending the modulation of charge carriers' behaviour induced by multieffects will help improve the performance of the devices. (2) The lifetime of the multieffect coupled NGs needs to be investigated for practical applications. (3) Multifunctional materials are required to be optimized since they have a vital influence on the multieffect coupled NGs. (4) Although the multieffect coupled NGs exhibit strong ability for harvesting multitype energies, their relative energy conversion is needed to be further promoted. (5) Routes towards reducing the manufacture cost are needed to be worked out. Nevertheless, with the deeper investigation of theory and improvements of materials and technology, multieffect coupled NGs are believed to be applied globally.

## Figures and Tables

**Table 1:** The characteristic comparison of self-powered photodetectors on the basis of diverse multieffect coupled NGs [20, 35, 43, 44, 62–70].

Materials	Working mechanism	Wavelength	Intensity	Responsivity	Rise time	Fall time	Ref.
BFO	Thermo-photovoltaic effect	365 nm	3.0 mW cm^−2^	6.0 × 10^−4^ A W^−1^	10.8 s	0.6 s	[20]
CH_3_NH_3_PbI_3_/ZnO	Pyro-photovoltaic effect	—	1.9 × 10^−5^ W cm^−2^	26.7 mA W^−1^	53 *μ*s	63 *μ*s	[35]
BTO	Pyro-photovoltaic effect	405 nm	209.8 mW cm^−2^	10^−7^ A W^−1^	0.6 s	0.5 s	[43]
BFO	Pyro-photovoltaic effect	450 nm	65 mW cm^−2^	—	0.5 s	0.8 s	[44]
p-si/n-ZnO	Pyro-photovoltaic effect	422 nm	—	79.9 mA W^−1^	0.6 ms	0.5 ms	[66]
ZnO	Pyro-photovoltaic effect	325 nm	3.8 × 10^−5^ W cm^−2^	1.25 m A W^−1^	318.9 ms	780.3 ms	[67]
CdS/P3HT	Piezo-photovoltaic effect	365-780 nm	—	—	<0.2 s	<0.2 s	[68]
GaN	Piezo-photovoltaic effect	400 nm	35 mW cm^−2^	11.6 mA W^−1^	<0.1 s	<0.1 s	[69]
ZnO/Spiro-MeOTAD	Piezo-photovoltaic effect	390 nm	3 mW cm^−2^	17 mA W^−1^	~200 *μ*s	~950 *μ*s	[70]
